# Source Authentication for Code Dissemination Supporting Dynamic Packet Size in Wireless Sensor Networks †

**DOI:** 10.3390/s16071063

**Published:** 2016-07-09

**Authors:** Daehee Kim, Dongwan Kim, Sunshin An

**Affiliations:** 1 Department of Electronics Engineering, Korea University, Seoul 02841, Korea; dhkim@dsys.korea.ac.kr (D.K.); sunshin@dsys.korea.ac.kr (S.A.); 2 Division of Network Business, Samsung Electronics Co., Ltd., Suwon 16677, Korea

**Keywords:** code dissemination, wireless sensor networks, dynamic packet size, source authentication

## Abstract

Code dissemination in wireless sensor networks (WSNs) is a procedure for distributing a new code image over the air in order to update programs. Due to the fact that WSNs are mostly deployed in unattended and hostile environments, secure code dissemination ensuring authenticity and integrity is essential. Recent works on dynamic packet size control in WSNs allow enhancing the energy efficiency of code dissemination by dynamically changing the packet size on the basis of link quality. However, the authentication tokens attached by the base station become useless in the next hop where the packet size can vary according to the link quality of the next hop. In this paper, we propose three source authentication schemes for code dissemination supporting dynamic packet size. Compared to traditional source authentication schemes such as μTESLA and digital signatures, our schemes provide secure source authentication under the environment, where the packet size changes in each hop, with smaller energy consumption.

## 1. Introduction

Code dissemination is a main building block of reprogramming which allows over-the-air software updates in wireless sensor networks (WSNs). Since WSNs are mostly deployed in unattended and hostile environments, secure code dissemination is essential to prevent attackers from injecting malicious code into the WSN. There have been a lot of works on secure code dissemination [1,2,3,4,5,6,7,8,9,10,11,12,13] whose main goal is to provide *source authentication*, which implies that each sensor node must be able to assure that the received code image is really sent by a base station (BS) and not modified in transit. They attain this goal by applying existing source authentication schemes, such as μTESLA [14], digital signatures and the hash chains, into code dissemination.

Recently, a few cross-layer approaches using link estimation have been suggested to improve energy efficiency in WSNs. DPLC [15] provides a lightweight dynamic packet length control scheme based on an accurate link estimation method which leads to low transmission overhead. ECD [16] is a code dissemination scheme supporting dynamic packet size and accurate sender selection based on link quality. Plena [17] is a packet length adaptation scheme using error estimating codes. Theses dynamic packet size control schemes can significantly improve the energy efficiency of code dissemination. However, it is not easy to guarantee source authentication for these schemes since the packet size can be changed in each hop depending on the link quality. All well-known existing source authentication schemes for WSNs, such as μTESLA, digital signatures and the hash chains, support only fixed-size packets in every hop during code dissemination.

In this paper, we propose three source authentication schemes for code dissemination supporting per-hop dynamic packet size, which implies that original packets in the first hop can be split or merged in the other hop depending on the link quality. Our paper starts from the fact that code dissemination schemes supporting dynamic packet size can optimize the energy efficiency by reducing transmission overhead based on the link quality. Under this environment, our schemes reinforce the security, especially source authentication which guarantees both authenticity and integrity for a new code image. According to our survey on existing works, source authentication for code dissemination supporting dynamic packet size has never been researched earlier. In this paper, three source authentication schemes are suggested as follows:

- Simple packet aggregation (SPA)
- Message authentication codes (MACs) based source authentication (MBSA)
- Bloom filter based source authentication (BFBSA)

These schemes are very efficient in terms of computation overhead since they mainly perform lightweight hash operations and require only one public key cryptographic operation during code dissemination. In addition, our schemes can work with any code dissemination scheme supporting dynamic packet size. The main contributions of the paper are as follows.

- We identify the problem of existing source authentication schemes which does not support code dissemination with per-hop dynamic packet size. According to our survey on existing works, our work is the first attempt to delve into source authentication for code dissemination with dynamic packet size.
- We propose three source authentication schemes to address the proposed problem with smaller energy consumption. We accomplish our objective by combining a variety of cryptographic functions such as the hash functions, digital signatures, and the Bloom filter.
- We analyze our work in terms of security and performance. More specifically, we discuss the authenticity, resilience to node capture attacks and denial-of-service (DoS) attacks in detail, and then present performance analysis with regard to computation and communication overhead.

Compared with our previous work [18], this paper has three major extensions which include the extensive survey on related works, the clear definition of the problem, and the detailed analysis on computation and communication overhead of our work. The rest of the paper is organized as follows: in Section 2, we describe the related works in detail. Section 3 defines the problem to be resolved in this paper. Some preliminaries prior to our proposal are presented in Section 4. In Section 5, we propose three source authentication schemes in detail. After analyzing our schemes in Section 6 and Section 7, Section 8 concludes the paper.

## 2. Related Work

**Figure 1:**
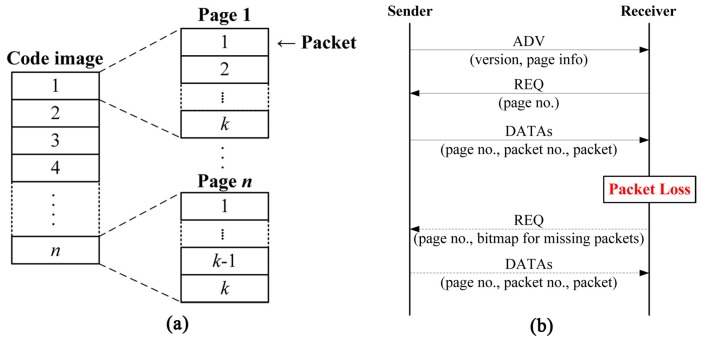
An illustration of Deluge: (**a**) The structure of pages and packets; (**b**) The operation of three-way handshake.

One of the most popular code dissemination protocols in WSNs is Deluge [19], which is the de facto standard [1,4,5,10,12,13] and has already been implemented in TinyOS. As shown in Figure 1a, Deluge splits the code image into fixed-size pages, each of which is then divided into fixed-size packets for pipelining and spatial multiplexing. Deluge uses a three-way handshake to transmit these packets for reliable delivery. The sender first broadcasts an advertisement (ADV) which includes the current version of the code image and page information. Upon receiving the ADV, the receiver sends a request (REQ) back to the sender for the specific page. The sender then begins to transmit data packets (DATAs) for the requested page. If the receiver does not receive all packets within the page successfully, it asks the sender for lost packets by sending a REQ indicating the specific lost packets within the page. Figure 1b shows the operation of Deluge. Even though Deluge itself supports fixed-size packets only and does not take security into account, most of non-secure and secure code dissemination protocols are built on the basis of Deluge.

To enhance the energy efficiency, a few works on dynamic packet size control have been introduced in the field of WSNs. There is definitely a tradeoff between using large-size packets in the good channel condition to reduce the header overhead and using small-size packets in the bad channel condition to reduce packet error rates (PER). DPLC [15] is an iterative algorithm to find an optimal packet size using the lightweight and accurate link estimation method. ECD [16] adapts the packet size depending on the 1-hop link quality for efficient code dissemination. Plena [17] is a packet size adaptation scheme using error estimating codes for WSNs. All of these schemes significantly conserve energy by adapting the packet size to the link quality. By integrating these schemes with existing code dissemination protocols such as Deluge, we can build an adaptive code dissemination scheme which dynamically adjusts the packet size depending on the link quality, thereby reducing the energy consumption. As it will be clarified in Section 3, our goal is to provide source authentication for these new kinds of code dissemination schemes where the packet size can be different in each hop.

Source authentication in WSNs has been addressed by μTESLA [14] and digital signatures. μTESLA provides source authentication by using a one-way key chain and a delayed key disclosure technique. Since the BS only knows the current key which is disclosed after broadcasting messages, the receivers can assure that the messages are from the real BS by verifying the one-way key as *h*(*K_i_*_+1_) = *K_i_* where *K_i_* and *K_i_*_+1_ are a previous key and a current key, respectively. μTELSA is very efficient since it only performs symmetric key cryptographic operations. However, μTESLA have a critical shortcoming that it must buffer all messages until the key is distributed, thus it is subject to DoS attacks which fill the buffer of the receiver by flooding it with false messages. Furthermore, since the intermediate nodes do not know the key, they cannot adjust the packet size which means that μTESLA only supports fixed-size packets in each hop. 

Digital signatures provide source authentication by simply signing each message with the private key of the BS. The receivers can assure the authenticity by verifying the received message with the public key of the BS. In contrast to μTESLA, digital signatures provide immediate authentication, thus they are strong to DoS attacks since there is no need to buffer messages. However, digital signatures are still heavy to resource-constrained sensor nodes even though Wander et al. [20] showed that public-key cryptography (PKC) is feasible on the sensor node by using elliptic curve cryptography (ECC). Using one digital signature for the entire code image as presented in [21] can be a candidate for providing source authentication because it not only addresses the computation overhead of digital signatures but also supports variable-size packets. However, since the digital signature is computed over the entire code image, it cannot be verified until all packets in the code image are completely received, which leads to the following problems. First, if the verification fails, all packets must be discarded because the receiver cannot identify which each packet is correct or not. This incurs the waste of the energy which is the most important resource in WSNs. Second, an attacker can easily disable the receiver by making the buffer of the receiver full by sending a lot of spoofed messages since the receiver does not identify each packet and thus keep all messages to make up the entire code image. 

**Figure 2:**
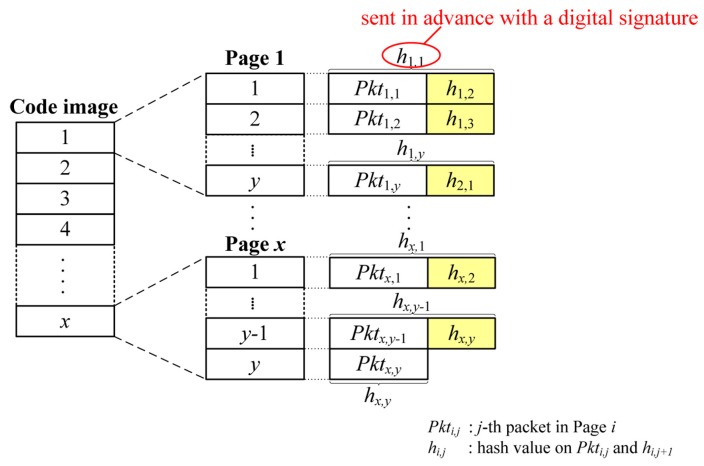
An example of Secure Deluge.

Due to the property that a code image is available prior to dissemination, existing secure code dissemination schemes in WSNs provide source authentication in a different way from μTESLA and digital signatures by using hash functions such as the hash chain or the Merkle hash tree. Secure Deluge [1] uses a hash chain as depicted in Figure 2. After the code image is divided into packets in the same way as Deluge, the hash is computed from the last packet and appended to the end of the previous packet. The procedure is repeated until the hash on the first packet is computed. The first hash (*h*_1,1_ in Figure 2) is then transmitted in advance after signing with a private key of the BS. Upon receiving the message, the receiver verifies the digital signature and stores the hash in order to use for authenticating the subsequent packet. The subsequent packet is verified by comparing the previously received hash and the hash on the currently received packet as *h*_1,1_ = *h*(*Pkt*_1,1_|*h*_1,2_). Secure Deluge is highly efficient since it uses only one digital signature and inexpensive hash functions. Secure Deluge also provides immediate authentication unlike μTESLA, and it has lower overhead than other existing secure code dissemination schemes because other existing secure code dissemination schemes have additional overhead such as the Merkle hash tree and one-way key chains for enhanced features such as confidentiality and loss-resilience. However, Secure Deluge is still vulnerable to DoS attacks in case of out-of-order packet delivery and does not provide source authentication for the variable-size packets.

Seluge [2] provides immediate authentication for code dissemination packets using a Merkle hash tree which includes the hash images of packets in page 1. The packets in page 1 are authenticated by the hash images of the Merkle hash tree, and the subsequent packets are authenticated by the hash chain like Secure Deluge. Sluice [3] is similar to Secure Deluge except that Sluice uses the page-level hashes while Secure Deluge uses the packet-level hashes. However, the page-level hashes are more vulnerable to DoS attacks than the packet-level hashes since packets must be buffered for making up a page. Tan et al. [4] provides not only immediate authentication using the hash chain but also confidentiality by encrypting each packet with a session key derived from the hash chain. LR-Seluge [5] enhances loss resilience on the basis of Seluge by using a fixed-rate erasure code. DiCode [6] is almost the same as Seluge but it supports the distributed control for code dissemination which means that multiple authorized network users are allowed to update a code image without involving the BS. Tan et al. [7] uses multiple one-way key chains instead of the hash chain to provide immediate packet authentication. It does not use even one PKC operation, but it incurs key distribution overhead and the communication overhead due to large packet size. SDRP [8] is almost identical to DiCode except that SDRP uses identity-based cryptography rather than RSA for authenticating the Merkle hash tree. Deng et al. [9] also takes a similar approach to Seluge by using the Merkle hash tree. Bohli et al. [10] provides an efficient source authentication by combining the Merkle hash tree and the hash chain. Flexicast [11] provides an energy-efficient authentication through authenticated fingerprints and network-wide attestation. Chen et al. [12] provides source authentication by using the Merkle hash tree and provides confidentiality through effective XORs coding and multiple one-way hash chains. SecNRCC [13] provides an immediate authentication with confidentiality consideration by combining the hash tree and the one-way key chain. SIMAGE [22] is a secure code dissemination which adapts to the link quality through dynamic packet sizing. Even if SIMAGE provides confidentiality and integrity between neighboring nodes, it does not provide source authentication. All of these schemes, which we have discussed in this section, do not provide source authentication for code dissemination supporting dynamic packet size in each hop. In contrast to these existing schemes, our works provide efficient source authentication for code dissemination supporting per-hop dynamic packet size.

## 3. Problem Definition

This paper is motivated by recent works on dynamic packet size control in WSNs which tries to minimize the energy consumption by dynamically adapting packet size depending on the link quality [15,16,17,22,23]. Large packets can improve the energy efficiency by keeping the overhead of the header low, but at the same time large packets can reduce the energy efficiency by raising PER. In contrast, the use of small packets leads to low PER, but is not good in terms of the header overhead. Therefore, these works adjust the packet size appropriately depending on the link quality.

**Figure 3:**
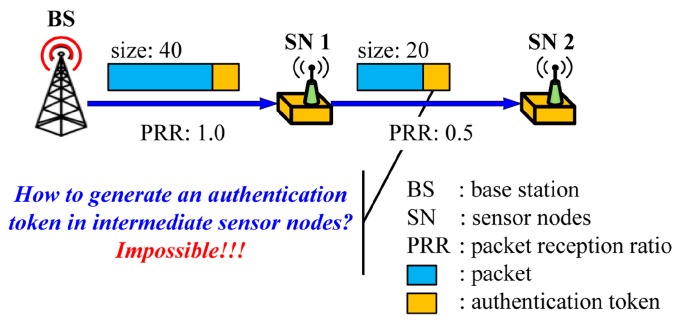
The problem of existing source authentication schemes in code dissemination supporting dynamic packet size.

Another important aspect of code dissemination is to guarantee the authenticity of the distributing node (e.g., BS) and the integrity of the code image which is normally ensured by the authentication tokens, such as MACs and digital signatures, attached to each packet by the source node. It is important to note that source authentication, not intermediate nodes authentication, is required during code dissemination since the sensor nodes are vulnerable to node capture attacks. When applying existing source authentication schemes into code dissemination supporting dynamic packet size, a new problem arises as shown in Figure 3 since the packet size can be different in each hop.

The BS first determines the packet size on the basis of the link quality between the BS and the sensor node 1. Once the packet size is determined, the BS generates an authentication token which provides the authenticity and the integrity of the packet, and sends the token with the packet to the node 1. Upon receiving the packet, the node 1 verifies the authentication token using a shared key with the BS or a public key of the BS according to the used cryptography. To forward the packet to the node 2, the node 1 determines the packet size depending on the link quality between the node 1 and the node 2. In this case where the optimal packet size is different from that of the first hop as shown in Figure 3, the node 1 cannot generate a valid authentication token of the BS for the packet with the optimal size since the key for generating an authentication token is only known to the BS.

Therefore, we define the problem to be resolved in this paper as “providing source authentication under the environment where the packet size changes in each hop during code dissemination in order to improve the energy efficiency.”

## 4. Preliminaries

### 4.1. Network Model for Code Dissemination

We assume that the underlying code dissemination scheme is Deluge. Additionally, we assume that the code dissemination scheme employs a dynamic packet size control scheme which adjusts a per-hop packet size based on the link quality in order to enhance energy efficiency such as DPLC, ECD, and Plena. Note that our schemes can be integrated with any kind of Deluge-based code dissemination schemes where the packet size is dynamically adjusted in each hop. For example, the scheme in [23] can be employed to obtain an optimal packet size depending on PER which is estimated by the medium access control layer acknowledgements. Although both the medium access control and the message authentication code are abbreviated as MAC, we only use MAC as the message authentication code to avoid confusion in the paper. Finally, it is important to note that our work can be applied to code dissemination with the fixed packet size as well as the dynamic packet size.

A BS, which is a source in our work, is assumed to be responsible for securely disseminating a code image to all sensor nodes in the WSN over multihop communications and always trustworthy. Each node then authenticates the code image by verifying the authentication tokens. All cryptographic primitives, including hash functions, MACs and digital signatures, are assumed to be secure. We assume that an attacker’s goal is to inject a malicious code into the WSN in order to get the information of interest or disable the WSN. Furthermore, the attacker is assumed to be able to compromise sensor nodes physically, but not infinitely.

### 4.2. Bloom Filter

**Figure 4:**
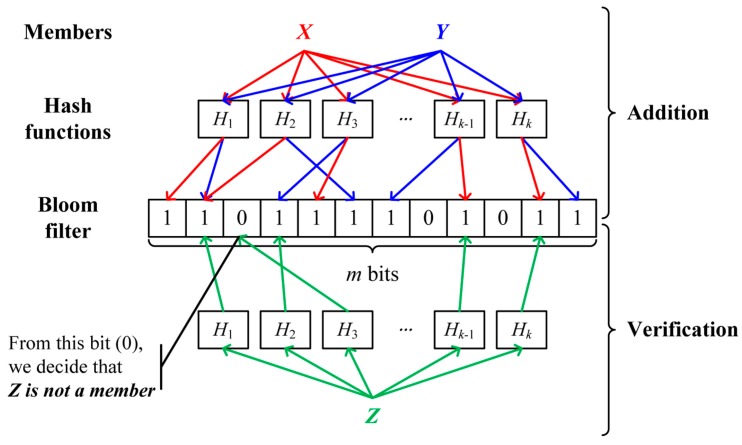
An example of the Bloom filter.

The Bloom filter [24] is a space-efficient probabilistic data structure used to examine the membership of an element in the set. The Bloom filter consists of a bit array of *m* bits, initially all set to 0, and *k* different hash functions used to map an element into one of positions in a bit array with a random uniform distribution. When adding an element to the Bloom filter, *k* hashes of an element are calculated using *k* hash functions and the bits on the position of *k* hashes are set to 1. To test whether an element is a member of the set, *k* hashes of an element are computed with *k* hash functions. If any bit on the position of *k* hashes is 0, the element is definitely not a member of the set. If all bits are 1, the element is regarded as a member of the set. Unfortunately, a false positive, indicating that a non-member element can pass the membership test, can occur due to the limited size of the Bloom filter and the duplicate occurrence of hash functions between different elements. Figure 4 illustrates a Bloom filter.

## 5. Proposed Source Authentication Schemes

In this section, we propose three source authentication schemes for code dissemination supporting dynamic packet size. Source authentication, the most significant security requirement for code dissemination in WSNs, ensures that a new code image is really sent by the BS and not altered in transit. Note that, for simplicity, we explain our schemes in the first hop which is between the BS and the neighbor node but this procedure continues to all sensor nodes in the WSN over multihop communications.

### 5.1. Simple Packet Aggregation (SPA)

**Figure 5:**
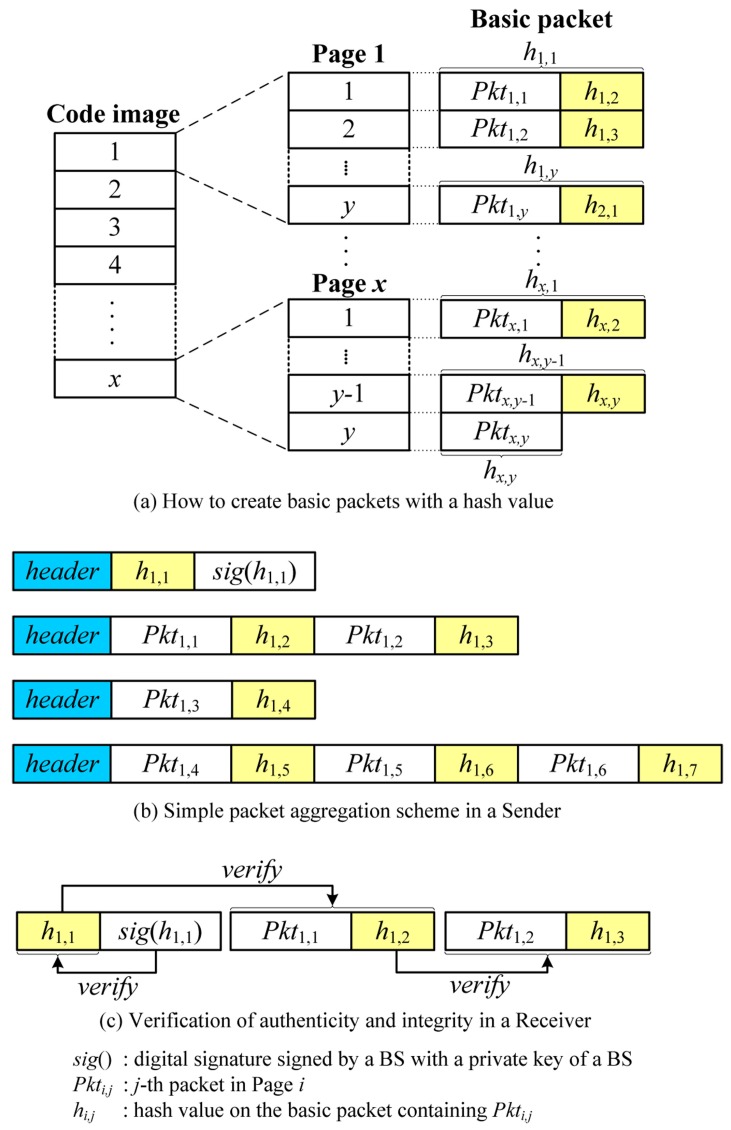
An example of SPA.

The simplest way to support source authentication for code dissemination with variable packet size is to simply aggregate packets as depicted in Figure 5. We employ a Secure Deluge [1] which uses a hash chain. In this scheme, a source can precompute a hash value of the next packet and embed it in the current packet since a new code image is built prior to the transmission. Then, a receiver can authenticate the next packet using the previously received hash value.

When a new code image is available, a BS splits it into pages which are further divided into packets with a fixed size. All packets are indexed sequentially, and a hash value is calculated using the next packet on the reverse order. The hash value is then appended to the end of the current packet, thereby forming a *basic packet* which is the basic unit to be transmitted as follows:

(1) $$BP_{i,j}\;=\;\{\begin{array}{ll} Pkt_{i,j}\;||\;h_{i,j+1}, & h_{i,j+1}\;=\;H(BP_{i,j+1}),\;i\in [1,x],\;j\in [1,y-1]\\ Pkt_{i,j}\;||\;h_{i+1,1}, & h_{i+1,1}\;=\;H(BP_{i+1,1}),\;i\in [1,x-1],\;j=y\\ Pkt_{i,j}, & i\;=\;x,\;j=y \end{array}$$

where *Pkt_i,j_* is a *j*-th packet in Page *i* and *h_i,j_* is a hash value on the basic packet containing *Pkt_i,j_*. *x* and *y* are the number of pages in the code image and the number of packets in one page, respectively. Finally, the hash of the first basic packet (*h*_1,1_) is signed by the BS with the BS’s private key to ensure source authentication. Figure 5a shows how to create basic packets with a hash value.

Once basic packets are ready, the BS first sends a hash value on the first basic packet (*h*_1,1_) with a digital signature (*sig*(*h*_1,1_)) to a neighbor node. The receiver first verifies the digital signature of the hash using a BS’s public key preloaded in each sensor node prior to deployment. If the digital signature is valid, the receiver stores the hash value which is used to authenticate the subsequent basic packet. The BS now transmits packets to the neighbor node by aggregating basic packets depending on the link quality as follows:

(2) $$BS\to *:\;[header||BP_{i,j}||BP_{i,j+1}||\cdot \cdot \cdot ||BP_{i,j+n}]$$

where *n* depends on the link quality. It is worth noting that the *BS* can send basic packets without aggregation in severe channel condition. Upon receiving an aggregated packet, the receiver splits it into the original basic packets. This is easy because each sensor node knows the length of a basic packet. After calculating the hash value on the first basic packet, the receiver compares it with the previously received hash value. Denoting the previously received hash value and the currently received basic packet by *h_i,j_* and *BP_i,j_*, the basic packet is considered valid if:

(3) $$h_{i,j}\;==\;H(BP_{i,j})\;,\;i\in [1,\;x],\;j\in [1,\;y]$$

where *H* is a hash function. If the hash values are the same, the basic packet is authenticated and the receiver assures that it comes from the real BS and is not modified during transmission. Finally, the hash value in the basic packet is stored to be used to authenticate the next packet. In the same way, all next packets can be verified. When all packets in one page are received, the receiver becomes a new sender. A new sender can send a packet by aggregating basic packets depending on the link quality. Figure 5b,c illustrates the process of aggregation and verification. The SPA scheme is very simple, but has the hash overhead per basic packet and supports only the multiples of the size of a basic packet.

### 5.2. MAC Based Source Authentication (MBSA)

MBSA makes use of peer-to-peer MACs to support any variable-size packets. However, MACs do not provide authenticity of the BS, which means that MACs authenticate the corresponding node only rather than the BS. To provide an authenticity of the BS, we employ a hash chain per page. Note that the previous SPA scheme uses a hash chain per packet.

**Figure 6:**
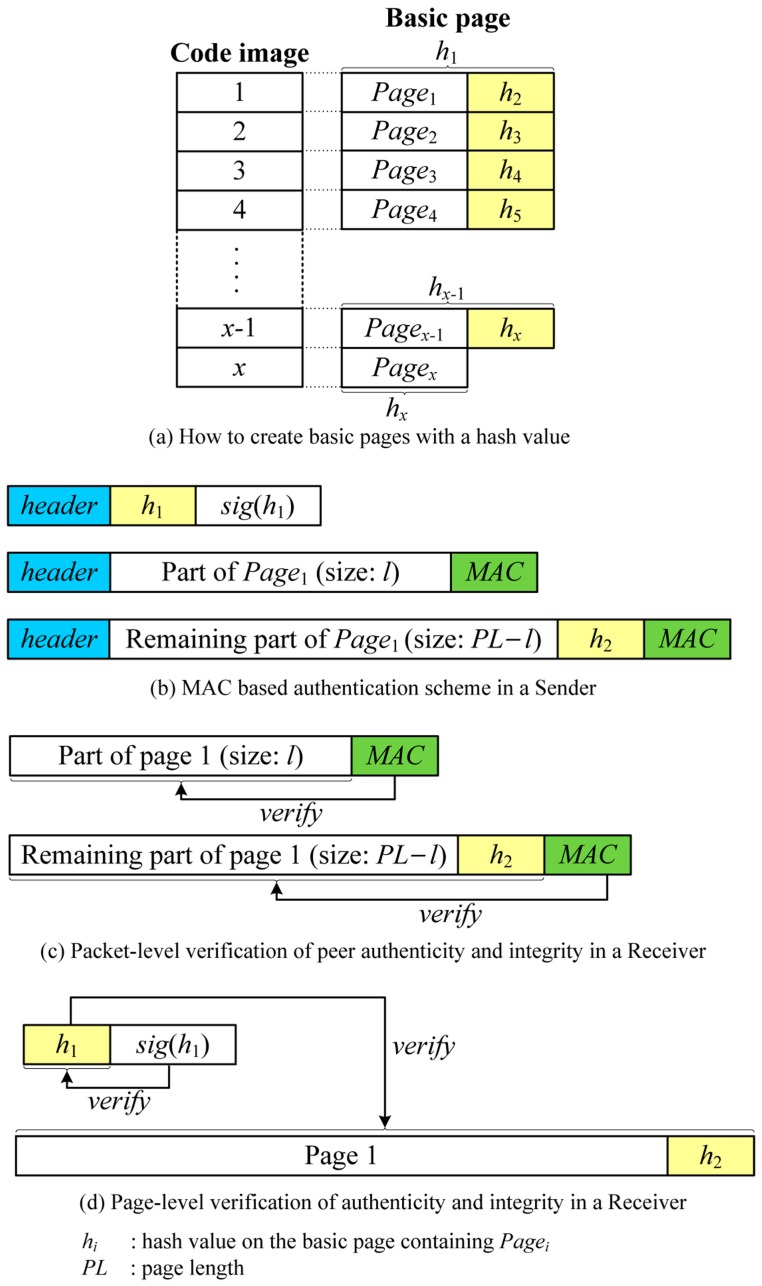
An example of MBSA.

In this scheme, a new code image is split into pages only unlike Deluge, after which all pages are indexed sequentially. Similar to the SPA scheme, a hash value on the next page is calculated and appended to the end of the page for the entire image, which together form a *basic page* as follows:

(4) $$BPage_{i}\;=\;\{\begin{array}{ll} Page_{i}\;||\;h_{i+1}, & \;h_{i+1}\;=\;H(BPage_{i+1}),\;i\in [1,x-1]\\ Page_{i}, & \;i=x \end{array}$$

where *Page_i_* is a *i*-th page and *h_i_* is a hash value on the basic page containing *Page_i_*. *x* is the number of pages in the code image. Finally, the hash of the first page is signed by the BS with the BS’s private key. This is illustrated in Figure 6a.

When sending a new code image, the BS first sends a hash value on the first basic page (*h*_1_) with a digital signature (*sig*(*h*_1_)) to a neighbor node. The receiver verifies the digital signature of the hash using a BS’s public key and keeps the hash value which is used to authenticate the subsequent basic page. The BS then decides the packet size depending on the link quality and attaches a MAC to the end of the packet using a symmetric key between the BS and the receiver. It is important to note that each node is assumed to have a symmetric key with neighbor nodes using any kind of key distribution schemes.

The resulting packet format for transmission in the MBSA scheme is expressed as follows:

(5) $$BS\to *:\;[header||payload_{i}||MAC(payload_{i})]$$

where *payload_i_* is the part of the basic page which can have any size. Figure 6b illustrates how to send packets using MACs in the sender. Upon receiving a packet, the receiver node first verifies the attached MAC. If it is not valid, the packet is not from the sender, thus discarded. If the packet is successfully verified as valid, it is parsed and kept in the internal buffer for making up a page. Once all packets in one page are received, the hash of the total page is calculated and compared to the hash value included in the previous page. The entire page is considered authentic if:

(6) $$h_{i}\;==\;H(BPage_{i}),\;i\in [1,x]$$

where *h_i_* is the previously received hash value for the *i*-th basic page and *BPage_i_* is the currently received *i*-th basic page. Figure 6c,d shows the procedure of packet-level verification using MACs and page-level verification using the hash chain, respectively. 

The MBSA scheme supports source authentication for fully variable packet size by combining both hop-by-hop authentication via MACs and source authentication via a page-level hash chain. Since this scheme needs only one MAC for variable-size packet, this scheme has less communication overhead than the SPA scheme. Moreover, it is more efficient in terms of energy consumption because it supports fully variable packet size so that it can be optimized for the link quality. The disadvantage of this scheme is that it can be vulnerable to node capture attacks. In this case, attackers can forge malicious packets with the valid MAC. However, it can be easily detected by the page-level hash chain.

### 5.3. Bloom Filter Based Source Authentication (BFBSA)

BFBSA takes advantage of a Bloom filter to verify the authenticity of packets. Like SPA, the BS splits a new code image into pages which are further divided into fixed-size packets called basic packets. It is important to note that basic packets in BFBSA is the same as packets while basic packets in SPA consist of packets and a hash value on the next basic packet. The BS then makes a *m*-bit length Bloom filter by applying *k* different hash functions to all basic packets as follows:

(7) $$BF[H_{n}(Pkt_{i,j})]\;=\;\{\begin{array}{cc} 1, & n\in [1,k],\;i\in [1,x],\;j\in [1,y]\\ 0, & \mathrm{otherwise} \end{array}$$

where *BF*[] is a *m*-bit array indicating a Bloom filter and *H_n_* is a hash function which maps packets into an integer with a range of 0 to *m*-1. 

When the Bloom filter is built, the BS broadcasts the entire Bloom filter to all sensor nodes together with a hash value on the first basic page (*h*_1_) after signing with a BS’s private key. It is important to note that *h*_1_ is used to verify authenticity and integrity of the page for defending against malicious packets due to false positive property of the Bloom filter. After each sensor node verifies a digital signature of the Bloom filter with a BS’s public key, it stores the Bloom filter to authenticate the subsequent packets.

The BS now sends a new code image to the neighbor nodes with variable-size packets, depending on the link quality as follows:

(8) $$BS\to *:\;[header||Pkt_{i,j}||Pkt_{i,j+1}||\cdot \cdot \cdot ||Pkt_{i,j+n}]$$

where *n* depends on the link quality, and the last packet can be fragmented. It is important to note that unlike previous schemes, there is no overhead for each packet such as MACs and hash values. Upon receiving a packet, the receiver node splits it into basic packets, each of which is then verified using the Bloom filter with *k* different hash functions. The basic packet is considered authentic if it satisfies the following equation:

(9) $$BF[H_{n}(Pkt_{i,j})]\;==\;1,\;\forall n\in [1,k]$$

**Figure 7:**
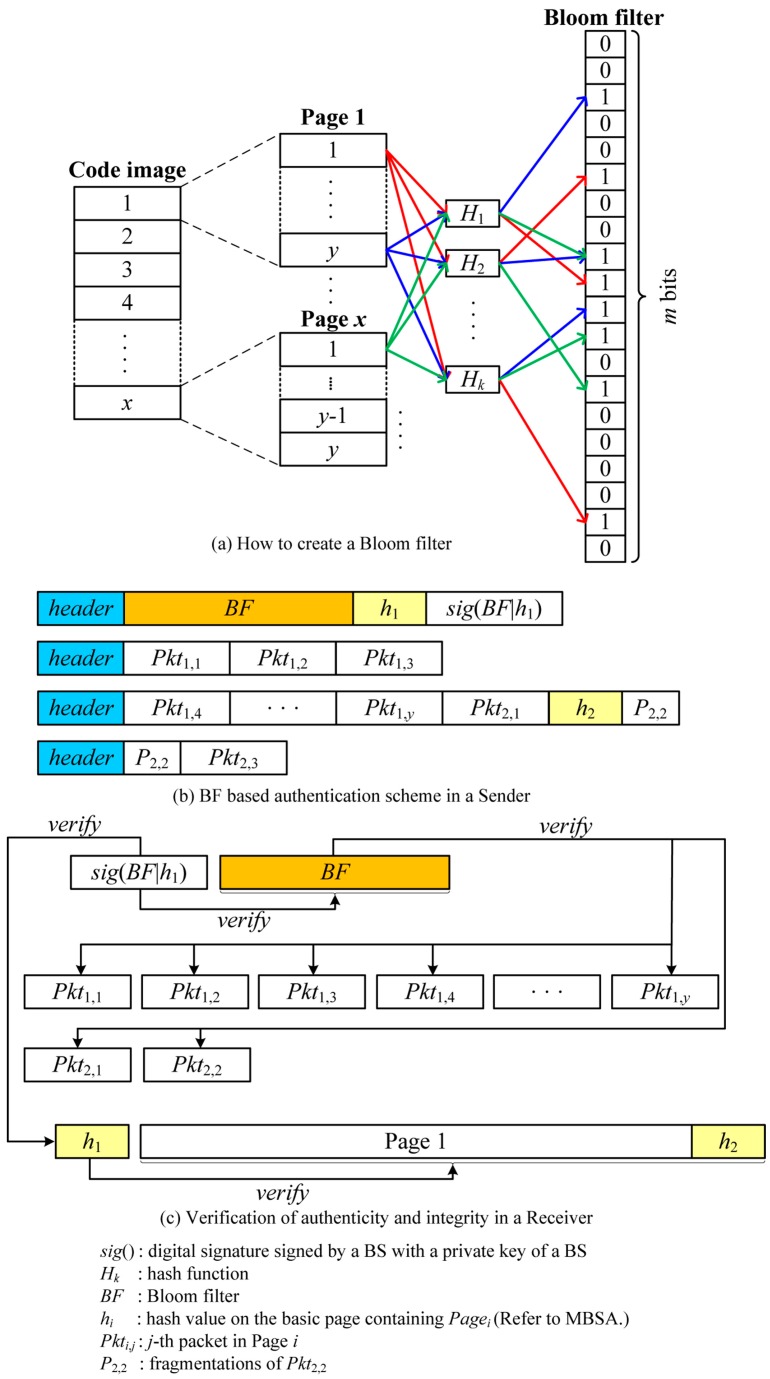
An example of BFBSA.

If it fails to pass the test, it definitely is not a valid packet. However, the authenticated packets might be forged packets due to false positive. To overcome this problem, we must adjust *k* and *m* appropriately which will be investigated in detail in Section 6. In addition, we add a hash value on the next page to the end of the last packet in each page. After receiving all packets in the page, the receiving node can verify the authenticity of the whole page with the hash value. This entire process is illustrated in Figure 7.

This scheme incurs storage and communication overhead for the Bloom filter but has no communication overhead in each packet. In addition, this scheme can support fully variable-size packets.

## 6. Security Analysis

In this section, we evaluate our three source authentication schemes in terms of security aspects, more specifically, authenticity, resilience to node capture attacks and DoS attacks.

### 6.1. SPA

Security of the SPA scheme depends on the security of hash functions and digital signatures. In general, hash functions are assumed to be secure which means that given *y*, it is computationally infeasible to find *x* such that *h*(*x*) = *y*. In addition, a BS is assumed to be always secure, thus the BS’s private key is never exposed to attackers. Hence, a digital signature signed by the BS is always secure which implies that any attacker cannot forge a valid digital signature of the BS. In the SPA scheme, every packet is authenticated by the previously received hash value. Since a hash function is assumed to be secure, the adversary cannot fabric a malicious packet with the same hash value as the previously received hash value. In other words, the SPA scheme can authenticate every packet securely. Now let’s think about the case where a sensor node is captured and controlled by an adversary, who can access and extract all the materials (e.g., *h_i,j_*) in the node. However, the only thing that the adversary can do is to drop packets since it is infeasible to fabric a malicious packet to have *h_i,j_*. If he modifies packets or injects malicious packets, it will be detected immediately through an invalid hash value. DoS attacks in the hash-chain based authentication scheme refers to the situation where the adversary try to make the buffer of receivers full by sending invalid packets repeatedly so that receivers get crippled. The SPA scheme is vulnerable to DoS attacks since it uses a hash chain containing the hash value of the next packet. If one packet is missing, all the subsequent packets cannot be authenticated and thus the buffer becomes full.

### 6.2. MBSA

Since the MBSA scheme uses a page-level hash chain, it can authenticate each page just as the SPA scheme authenticates each packet. However, this page-level authentication is vulnerable to DoS attacks because it has to keep all received packets to make up a page. In this scheme, DoS attacks can be circumvented by hop-by-hop authentication using MACs with a symmetric key. If any node without a symmetric key attempts to inject malicious packets, it will be detected by the MAC. Unfortunately, this scheme is susceptible to node capture attacks. Once a sensor node is compromised, all information, including all symmetric keys with neighbor nodes, is disclosed to the adversary. Therefore, the adversary can easily inject malicious packets to the neighbors. However, this can be prevented by the page-level hash (*h_i_*) since the adversary cannot fabricate a whole page which has *h_i_*. In summary, the MBSA scheme provides an immediate authentication using MACs, thus it is robust to DoS attacks as long as sensor nodes are not compromised. However, the MBSA scheme is weak to node capture attacks since compromised nodes can send forged packets to their neighbors, but the page-level hash constrains the effect within the page.

### 6.3. BFBSA

Security of BFBSA depends on the probability of false positive. Given the number of basic packets, *N*, and a bit array of the Bloom filter with *m* bits, the probability *p* of false positive, with optimal *k* = (*m*/*N*)ln 2, is as follows [25]:

(10) $$p\;=\;{\left(0.6185\right)}^{\frac{m}{N}}$$

**Figure 8:**
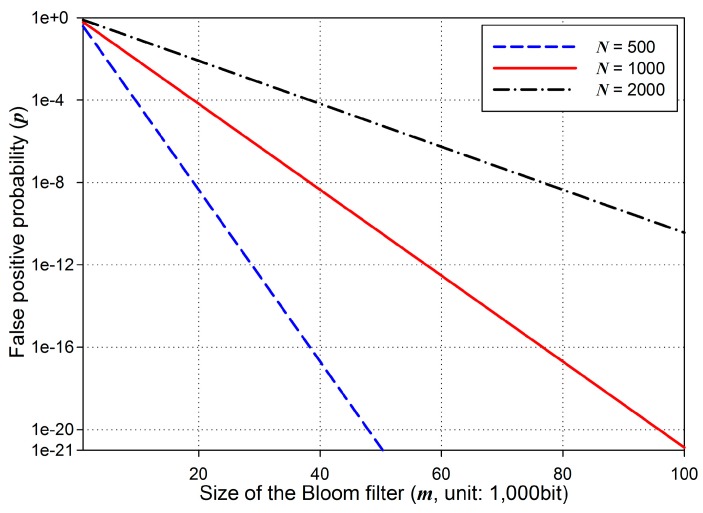
The probability of false positive in the Bloom filter.

Obviously, *p* decreases with lower *N* and higher *m* as shown in Figure 8. In code dissemination in WSNs, the number of basic packets, *N*, is predetermined according to the size of a new code image. Given *N*, the probability of false positive with regard to *m* is determined using (10). For example, we assume that *N* is set to 1000 packets. When *m* is configured as 1000 bits, *p* becomes 0.6185 which is very high. However, when *m* increases from 1000 bits to 10,000 bits, *p* becomes 0.0082 which is much less than 0.6185. Furthermore, when *m* is set to 100,000 bits, *p* is 1.36 × 10^−21^. This means that approximately 2^69^ elements have to be generated for an attacker to fabric a false positive packet. Therefore, the BFBSA scheme can authenticate every packet securely with a proper configuration of *m*. We set *m*/*N* to 92 targeting at the false positive probability of 6.36 × 10^−20^ which is assumed to be secure in [26]. In addition, even if a false positive occurs, it can be detected by the page-level hash. Regarding node capture attacks in BFBSA, compromised nodes cannot do anything but to discard packets since it is infeasible for them to build forged packets to pass the Bloom filter and the page-level hash. Moreover, BFBSA is resilient to DoS attacks since it provides an immediate authentication per packet.

## 7. Performance Evaluation

Since sensor nodes have very limited resources, various overhead must be minimized to reduce energy consumption. In this section, we evaluate our proposed three source authentication schemes in terms of computation overhead and communication overhead through an analytical approach. We then compare the results with Secure Deluge, which provides source authentication for fixed-size packets and has lower overhead than existing well-known secure code dissemination protocols as presented in Section 2.

### 7.1. Environment for Performance Evaluation

**Figure 9:**
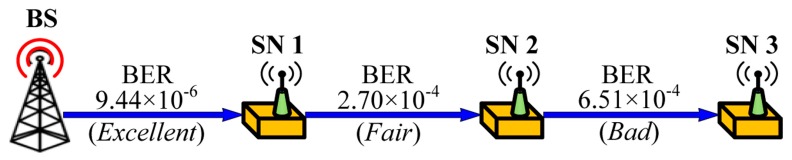
Configuration for the performance evaluation.

**Figure 10:**
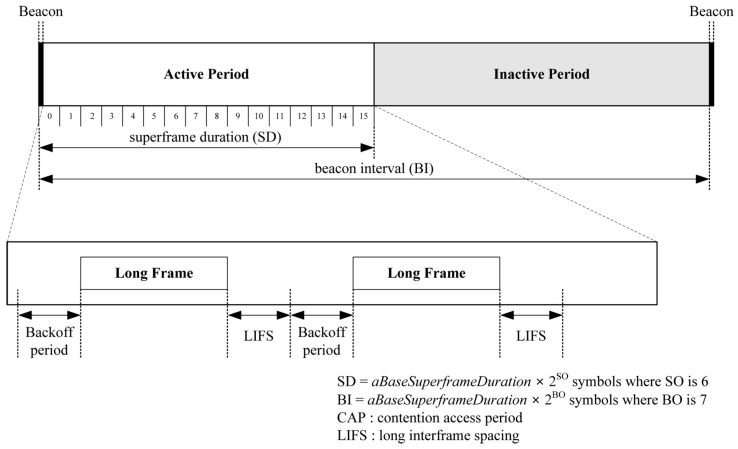
The structure of IEEE 802.15.4 superframe in the performance evaluation.

The goal of the performance evaluation is to show that our proposed schemes can reduce energy consumption when integrating with code dissemination supporting dynamic packet size according to the link quality. We assume a simple 3-hop topology with different link quality as shown in Figure 9, where a bit error rate (BER) is assigned to each link to indicate excellent (PRR: 0.99), fair (PRR: 0.75) and bad (PRR: 0.5) link quality when the packet size is 133 bytes that include a PHY header (6 bytes) and a maximum medium access control protocol data unit (127 bytes) in IEEE 802.15.4 [27] as shown in Figure 10. We assume that code dissemination is performed in sequential order, thus no collisions due to channel contention occur in the simple 3-hop topology. We employ IEEE 802.15.4 as the medium access layer where we use the beacon-enabled mode, and do not use the acknowledgement as shown in Figure 10. In order to take the practical environment into account, the duty cycle is set to 50% by setting the value of the *macSuperframeOrder* (SO) for the superframe duration (SD) to 6 and the value of the *macBeaconOrder* (BO) for the beacon interval (BI) to 7 as shown in Figure 10. As a result, the node transmits packets as a long frame in the active period while entering the sleep mode in the inactive period. The long frame is the IEEE 802.15.4 data frame where the size of medium access control protocol data unit (MPDU) is larger than 18 bytes, followed by a long interframe spacing (LIFS) period. We employ the energy model of TelosB [28], which is a commonly used sensor node [15,16,17], as follows.

(11) $$E\;=\;E_{rx}+E_{tx}+E_{mcu}+E_{idle}+E_{sleep}$$

For the sake of simplicity, we do not consider the energy consumption for the transition procedure among TX, RX, and off state, and assume that the CC2420 makes a transition to the off state immediately after completing data transmission. *E_rx_* and *E_tx_* are the energy consumed when the microcontroller unit (MCU) is on and the radio is RX or TX, *E_mcu_* is the energy consumed when the MCU is on and the radio is off, *E_idle_* is the energy consumed when the MCU is idle and the radio is off, and *E_sleep_* is the energy consumed when the node is in the sleep mode. In other words, *E_rx_* and *E_tx_* are the energy consumption for receiving and transmitting the code image, and *E_mcu_* corresponds to the energy consumption for performing cryptographic operations such as digital signatures, MACs and hashes. In addition, *E_idle_* is the energy consumption during the backoff period and the LIFS in the active period, and *E_sleep_* is the energy consumption in the inactive period in Figure 10. The energy consumption in each mode is computed by multiplying the time in each mode with the supply voltage and the current in each mode of TelosB which is shown in Table 1. For example, *E_tx_* for transmitting a 40-byte packet is (40 bytes × 32 μs) × 3 V × 19.5 mA = 74.88 μJ. 

**Figure 11:**
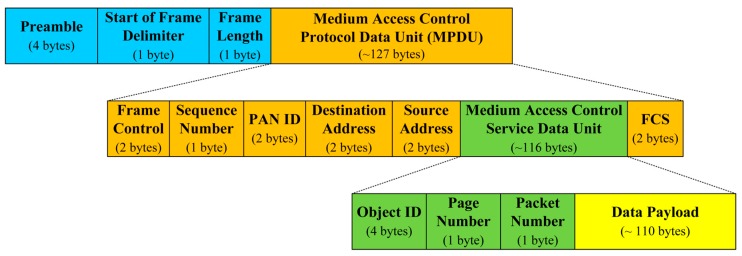
The format of IEEE 802.15.4 data frame.

We assume that the underlying code dissemination protocol is Deluge, and a page size and a packet size are configured to 1024 bytes and 22 bytes which are the default configurations of Deluge in TinyOS. The size of a code image to be disseminated is 10 kB, 20 kB and 30 kB. Additionally, the underlying code dissemination protocol is assumed to be able to optimize the size of the data payload in the medium access control service data unit in Figure 11 according to the given BER in terms of the transmission overhead (*TO*) which is defined as

(12) $$\begin{array}{l} TO\;(x)=\frac{Total\;amount\;of\;bytes\;to\;be\;transmitted}{Payload\;size(bytes)}\\ \;=\frac{(hdr+x)/PRR}{x}=\frac{hdr\;+\;x}{x\cdot {\left(1-BER\right)}^{8(hdr+x)}}\\ \mathrm{Find}\;x_{opt}\text{ to minimize }TO(x)\;\to \;\frac{d}{dx}TO(x_{opt})=0\\ x_{opt}=\{\begin{array}{ll} \frac{-4\cdot hdr\cdot \ln(1-BER)-\sqrt{{\left(4\cdot hdr\cdot \ln(1-BER)\right)}^{2}-8\cdot hdr\cdot \ln(1-BER)}}{8\cdot \ln(1-BER)} & if\;x_{opt}\;<\;110\\ 110 & if\;x_{opt}\geq 110 \end{array} \end{array}$$

where *x* is the size of the data payload in Figure 11, and *hdr* includes all headers in Figure 11 and all cryptographic overhead such as hashes and MACs. Since the total amount of the data payload, which is the size of the code image, is fixed in code dissemination, the total transmitted bytes decrease as *TO* becomes lower, thus reducing energy consumption. For the sake of simplicity, we assume that the optimal payload size, which minimizes *TO* in Equation (12), of each scheme in each link is predetermined by applying BER and *hdr* into Equation (12) as shown in Table 2. Each packet is lost in each hop with the probability of *PER* = 1 – *PRR* = 1 − (1 − *BER*)^8*L*^ where *L* is a packet size, including the data payload and the header, in bytes. The lost packet is retransmitted according to the procedure of Deluge as shown in Figure 1b. It is important to note that all kinds of Deluge messages such as the ADV, the REQ and DATAs are transmitted as a long frame in the active period in Figure 10 since the MPDU size of all Deluge messages is larger than 18 bytes. Other parameters for the performance evaluation are summarized in Table 1.

### 7.2. Computation Overhead

We consider computation overhead needed for each node to authenticate an entire code image. Computation overhead per node in each scheme is as follows:

(13) $$CP_{SecureDeluge}=C_{sig}\;+\;N_{pkt}^{basic}\cdot C_{hash}$$

(14) $$CP_{SPA}\;=\;C_{sig}\;+\;N_{pkt}^{basic}\cdot C_{hash}$$

(15) $$CP_{MBSA}\;=\;C_{sig}\;+\;N_{page}\cdot C_{hash}\;+\;2\cdot N_{pkt}^{tx}\cdot C_{MAC}$$

(16) $$CP_{BFBSA}\;=\;C_{sig}\;+\;N_{page}\cdot C_{hash}\;+\;N_{pkt}^{basic}\cdot k\cdot C_{hash}$$

where *C_sig_*, *C_hash_* and *C_MAC_* denote the computation overhead of digital signatures, hash operations and MAC operations, respectively. *N_page_* is the number of pages, which is determined as ⌈code  image  size / page  size⌉. $N_{pkt}^{basic}$ is the number of packets in the entire code image, which is computed as $N_{page}\times \left\lceil \text{page size }/\text{ packet size}\right\rceil$. $N_{pkt}^{tx}$ is the number of packets transmitted actually under the given link quality. *k* is the number of hash functions used in the Bloom filter. From Equations (13)–(16), we can derive the energy consumption due to computation overhead as follows:

(17) $$E_{mcu}^{SecureDeluge}=I_{mcu}\cdot V_{sup}\cdot T_{sig}+N_{pkt}^{basic}\cdot I_{mcu}\cdot V_{sup}\cdot T_{hash}$$

(18) $$E_{mcu}^{SPA}=I_{mcu}\cdot V_{sup}\cdot T_{sig}+N_{pkt}^{basic}\cdot I_{mcu}\cdot V_{sup}\cdot T_{hash}$$

(19) $$E_{mcu}^{MBSA}=I_{mcu}\cdot V_{sup}\cdot T_{sig}+N_{page}\cdot I_{mcu}\cdot V_{sup}\cdot T_{hash}+2\cdot N_{pkt}^{tx}\cdot I_{mcu}\cdot V_{sup}\cdot T_{MAC}$$

(20) $$E_{mcu}^{BFBSA}=I_{mcu}\cdot V_{sup}\cdot T_{sig}+N_{page}\cdot I_{mcu}\cdot V_{sup}\cdot T_{hash}+N_{pkt}^{basic}\cdot k\cdot I_{mcu}\cdot V_{sup}\cdot T_{hash}$$

**Figure 12:**
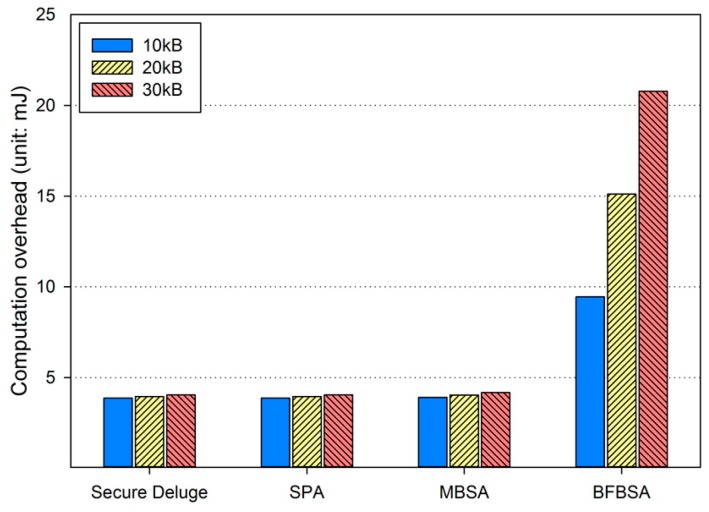
Computation overhead in bad link quality.

Since the computation overhead shows similar results in each link quality, we only present Figure 12 which shows the energy consumption for performing cryptographic operations in the bad channel condition. Obviously, computation overhead of the BFBSA scheme is the largest among all of schemes since BFBSA performs many more hash operations to verify the membership of each packet against the Bloom filter. It is worth noting that the computation overhead is constant in each hop regardless of the link quality because the lost packets can be retransmitted without computation. 

### 7.3. Communication Overhead

**Figure 13:**
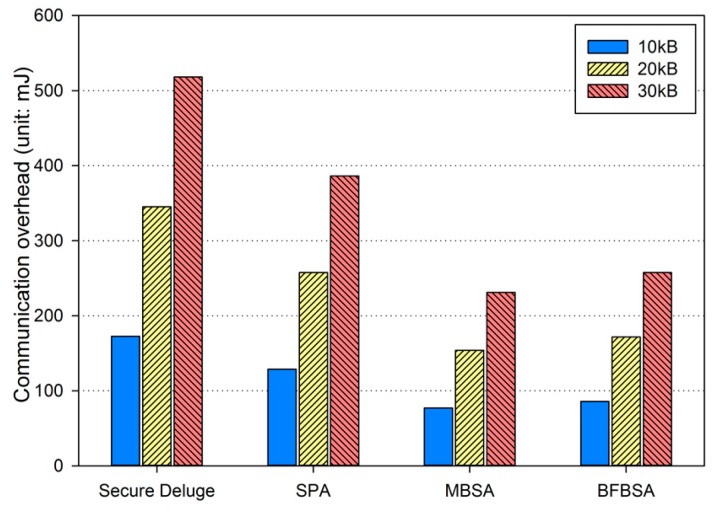
Communication overhead in excellent link quality.

**Figure 14:**
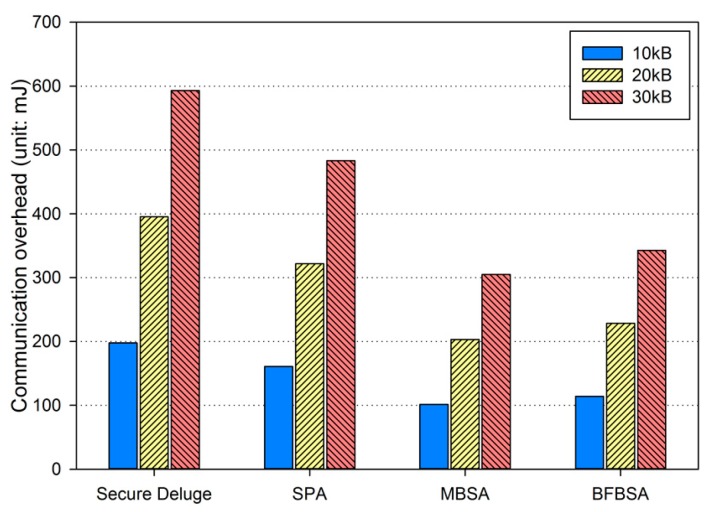
Communication overhead in fair link quality.

**Figure 15:**
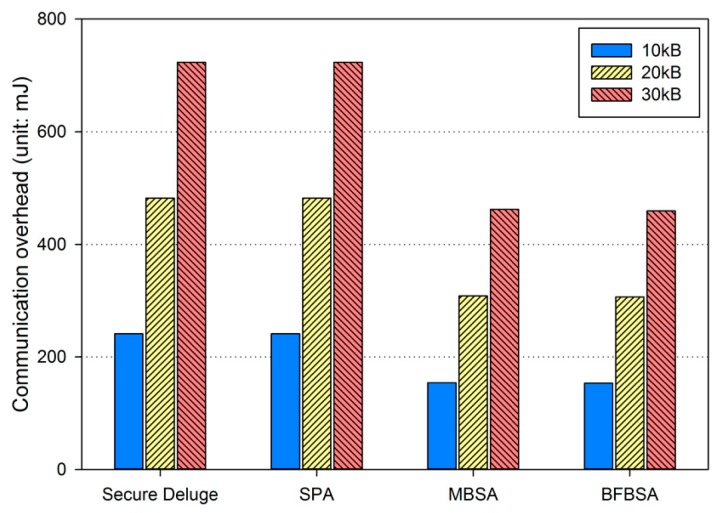
Communication overhead in bad link quality.

In this subsection, the energy consumed for sending a code image to the neighbor node are computed and then compared with Secure Deluge. We first investigate the total bytes transmitted for an entire code image since communication overhead is very important in that it is directly related to energy consumption. When PRR is 1 which implies that no packet loss happens, total transmitted bytes of each scheme is expressed as:

(21) $$CM_{SecureDeluge}=N_{pkt}^{basic}\cdot (L_{header}+L_{payload}+L_{hash})+L_{sig}+N_{page}\cdot (L_{ADV}+L_{REQ})$$

(22) $$CM_{SPA}=N_{pkt}^{tx}\cdot L_{header}+N_{pkt}^{basic}\cdot (L_{payload}+L_{hash})+L_{sig}+N_{page}\cdot (L_{ADV}+L_{REQ})$$

(23) $$CM_{MBSA}=N_{pkt}^{tx}\cdot (L_{header}+L_{MAC})+N_{page}\cdot (L_{page}+L_{hash})+L_{sig}+N_{page}\cdot (L_{ADV}+L_{REQ})$$

(24) $$CM_{BFBSA}=N_{pkt}^{tx}\cdot L_{header}+N_{page}\cdot (L_{page}+L_{hash})+L_{sig}+m+N_{page}\cdot (L_{ADV}+L_{REQ})$$

where *L_header_*, *L_payload_*, *L_hash_*, *L_sig_* and *L_MAC_* denote the length of a header, a payload, a hash value, a digital signature and a MAC, respectively. All Deluge packets are transmitted according to IEEE 802.15.4 presented in Figure 10. In other words, each packet is preceded by the backoff period and followed by the LIFS period. Therefore, the total energy consumption for sending one packet is computed as follows:

(25) $$\begin{array}{ll} E_{pkt} & =E_{backoff}+E_{tx}^{pkt}+E_{LIFS}\\ & =I_{idle}\cdot V_{sup}\cdot T_{backoff}+I_{tx}\cdot V_{sup}\cdot T_{byte}\cdot L_{pkt}+I_{idle}\cdot V_{sup}\cdot \;T_{LIFS} \end{array}$$

where *E_backoff_* and *E_LIFS_* is the energy consumption during the backoff period and the LIFS period, respectively. $E_{tx}^{pkt}$ is the energy consumption for transmitting a packet with the size of *L_pkt_*. In addition, the receiver node is assumed to be in the Rx state and thus the energy consumption can be computed as *I_rx_·V_sup_·T_rx_* where *T_rx_* is the duration over which the receiver node is in the RX state. Finally, *E_sleep_*, which is the energy consumption when the sender enters the inactive period in Figure 10, is calculated as *I_rx_·V_sup_·T_inactive_*. By considering all energy consumption not only for transmitting and receiving the code image but also for the backoff period, the LIFS period and the inactive period defined in IEEE 802.15.4 as shown in Figure 10, we obtained the energy consumption due to the communication overhead as shown in Figure 13, Figure 14 and Figure 15, each of which corresponds to the excellent, fair and bad link quality, respectively.

**Figure 16:**
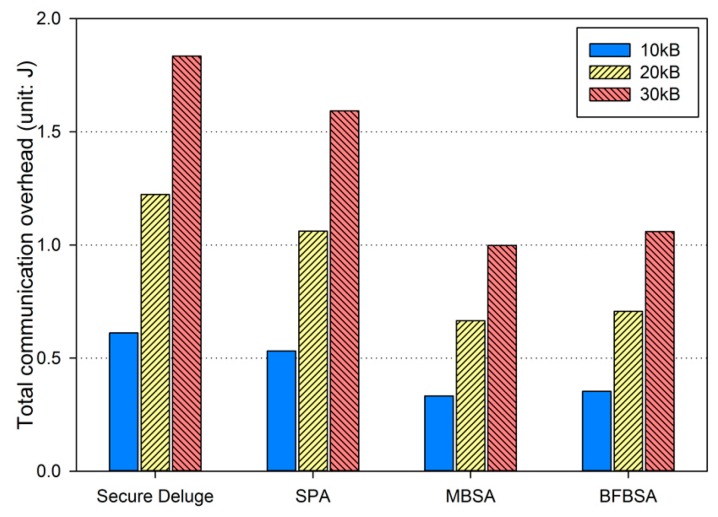
Total communication overhead.

In excellent link quality as shown in Figure 13, SPA, MBSA and BFBSA have lower communication overhead than Secure Deluge by approximately 25.4%, 55.4% and 50.3%. This is because Secure Deluge cannot adapt its packet size to the link quality even though larger packet reduces communication overhead in the excellent link quality. MBSA and BFBSA outperform SPA since MBSA and BFBSA can support fully variable packet size while SPA can only support the packet size of multiples of the basic packet. MBSA is better than BFBSA since BFBSA need to transmit a Bloom filter with the size of $\left\lceil N_{pkt}^{basic}\times (m/N)\right\rceil$ to guarantee a false positive probability of 6.36 × 10^−20^. In fair link quality as shown in Figure 14, SPA, MBSA and BFBSA outperforms Secure Deluge by approximately 18.6%, 48.6% and 42.2%. Except that the gap between Secure Deluge and proposed schemes decreases since a small-size packet in Secure Deluge results in higher PRR, Figure 14 is similar to Figure 13. In bad link quality as shown in Figure 15, Secure Deluge and SPA has the same communication overhead since the optimal packet size of each scheme is identical under bad link quality. MBSA and BFBSA has lower communication overhead than Secure Deluge by approximately 36.1% and 36.5%. In this case, the communication overhead of MBSA becomes larger than BFBSA because the amount of MAC overhead becomes larger as the packet size becomes small whereas the Bloom filter size is fixed regardless of the packet size. Finally, the total communication overhead, summing communication overhead in all three hops, is shown in Figure 16. As you can see, our schemes have less communication overhead than Secure Deluge regardless of the size of the code image. Among our three schemes, MBSA and BFBSA outperform SPA since they can support fully variable packet size. MBSA has lower communication overhead than BFBSA since BFBSA needs a large Bloom filter to reduce the false positive probability.

### 7.4. Total Energy Consumption

**Figure 17:**
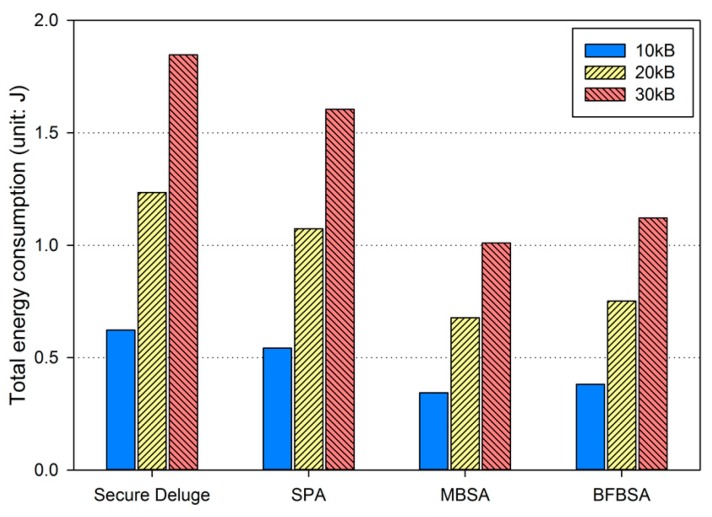
Total energy consumption.

Based on the results from previous subsections, we compute total energy consumption to show that our proposed schemes are more energy-efficient than Secure Deluge. In each hop, one node sends a code image which is received by the other node while only one computation is performed in the receiver node only. Figure 17 shows total energy consumption including communication and computation. SPA, MBSA and BFBSA consumes less energy than Secure Deluge by approximately 12.9%, 44.7% and 38.8%. This benefit become larger when the hop count increases and the link quality varies more dynamically. It is important to note that BFBSA consumes less energy than Secure Deluge despite high computation overhead because communication spends much more energy than computation.

### 7.5. Discussion

In this subsection, we discuss the results and clarify the limitations of our analysis. The goal of our performance evaluation is to show that our proposed schemes are more energy-efficient than existing schemes when integrating with adaptive code dissemination protocols which change the packet size depending on the link quality. To take into account the effect of the various link quality, we defined a simple 3-hop topology where each hop is assigned a static BER to represent different link quality. We additionally assumed that no collisions due to channel contention occur in order to focus on the channel error which is the dominant factor for the adaptive code dissemination. Finally, we adopted TelosB as a representative platform and the operation of the CC2420 wireless transceiver is simplified by ignoring the transition time among TX, RX, and off state. Under these assumptions and simplifications, we showed that our proposed schemes outperform Secure Deluge which is a well-known secure code dissemination scheme using fixed-size packets. As shown in Section 7.3, our proposed schemes work well in all kinds of link quality by adapting the packet size to the link quality while Secure Deluge uses small fixed-size packets of 22 bytes which is only good for the bad link quality. It is very important to note that our performance evaluation is conducted under the following assumptions, which will be considered carefully when applying to the real-world environment.

- the use of a simple 3-hop topology with static link quality
- the assumption of no collisions due to channel contention
- the use of TelosB
- the simplification about the behavior of the CC2420 wireless transceiver
- the use of Deluge

We used a simple 3-hop topology where each hop is assigned a static BER for the sake of simplicity. To achieve broader applicability of our work, we must further consider the wide range of network configurations and the effect of more realistic link quality by applying our schemes to the real-world testbeds with a variety of network configurations, which is one of our future works. We also assumed that no collisions due to channel contention happen. Even if the system performance such as energy consumption will be degraded by collisions due to channel contention, the proposed schemes will be influenced by the channel error more than collisions since they focus on the design about dynamic packet size depending on the channel conditions. However, the impact of collisions must be taken into account in order to obtain more accurate results in the real-world environment. We used TelosB as a representative platform because TelosB is slightly out-of-date [28] but it is still commonly used in WSNs [15,16,17]. Since the energy consumption can be different according to the platforms, the new performance evaluation is required before applying our schemes to the new platform. For simplicity, we ignored the transition time of the CC2420 wireless transceiver, which will have impact on the energy consumption of the real sensor nodes but it is difficult to reflect the operation of transitions of the CC2420 without experiments. Thus, we must perform the real-world experiment to obtain the exact energy consumption of each sensor node, which is included in our future works. Finally, our proposed schemes are based on Deluge which is the *de facto* standard code dissemination protocol in WSNs. Even though our work can provide source authentication for other code disseminations with slight modifications, we do not consider other code dissemination protocols in this paper since our works are originally targeted at WSNs where Deluge is the *de facto* standard.

## 8. Conclusions

In this paper, three source authentication schemes for code dissemination supporting dynamic packet size in WSNs have been proposed. According to our survey on existing works, source authentication for per-hop variable-size packets has never been researched earlier. Hence, our work gives a new opportunity to offer both energy efficiency and security by combining code dissemination with variable packet size and source authentication schemes together. Through the security analysis and the performance evaluation, we showed that all three schemes are superior to Secure Deluge in terms of security aspects and energy consumption which is summarized in Table 3, but each of our schemes has its own strengths and weaknesses. Hence, each of our schemes can be applied to the different environment. For example, if the possibility of node capture attacks is very low, MBSA is a best candidate due to its low energy consumption. On the other hand, if the required security level is high and each node has sufficient memory, BFBSA is a best candidate due to its robustness to attacks.

As discussed in Section 7.5, our work has limitations that do not reflect all of the realistic environments, including the use of a simple 3-hop topology with static link quality, the assumption of no collisions due to channel contention and the simplification about the behavior of the CC2420 wireless transceiver. To overcome those limitations, we will further enhance the proposed schemes by performing real-world experiments under the various network configurations.

## Figures and Tables

**Table 1:** Parameters for Performance Evaluation.

Parameter	Value (B: Bytes)	Description
*L_header_*	23 B	the header size (PHY: 6 B, Medium Access Control: 11 B, Deluge: 6 B)
*L_page_*	1024 B	the default page size of Deluge in TinyOS
*L_paylod_*	variable	the payload size for DATAs (the default payload size of Deluge in TinyOS: 22 B)
*L_ADV_*	29 B	the size of the ADV (PHY header: 6 B, Medium Access Control header: 11 B, Deluge: 12 B)
*L_REQ_*	32 B	the size of the REQ (PHY header: 6 B, Medium Access Control header: 11 B, Deluge: 15 B)
*L_hash_*	20 B	the hash size in SHA-1
*L_MAC_*	20 B	the MAC size in HMAC
*L_sig_*	40 B	the digital signature size in ECDSA-160
*m*/*N*	92	false positive of 6.36 × 10^−20^
*k*	64	(*m*/*N*)ln 2
code image size	10/20/30	unit: kB
*V_sup_*	3 V	the supply voltage in TelosB [28]
*I_tx_*	19.5 mA	the current when the MCU is on and the radio is TX in TelosB [28]
*I_rx_*	21.8 mA	the current when the MCU is on and the radio is RX in TelosB [28]
*I_mcu_*	1.8 mA	the current when the MCU is on and the radio is off in TelosB [28]
*I_idle_*	54.5 μA	the current when the MCU is idle and the radio is off in TelosB [28]
*I_sleep_*	5.1 μA	the current when the MCU is standby and the radio is off in TelosB [28]
SO/BO	6/7	the parameters to determine SD and BI
*T_SIFS_*	192 μs	the duration of SIFS (short interframe spacing)
*T_LIFS_*	640 μs	the duration of LIFS (long interframe spacing)
*T_backoff_*	*R* × 320 μs	the backoff period. *r* is a random value between 0 and 3
*T_inactive_*	983,040 μs	the duration of the inactive period when SO and BO is 6 and 7
*T_byte_*	32 μs	the time for transmitting one byte in TelosB
*T_long_*	variable	the long frame period over which a ADV/REQ/DATA is transmitted frame size (bytes) × byte transmission time (32 μs)
*T_hash_*	0.35 ms	the hash computation time in TelosB [29]
*T_MAC_*	0.71 ms	the MAC computation time in TelosB [29]
*T_sig_*	1.02 s	the signature verification time in TelosB [30]

**Table 2:** Optimal Payload Size.

BER	Scheme	*hdr* in Equation (12)	Payload Size (Bytes)
9.44 × 10^−6^ (Excellent)	Secure Deluge	43 (header: 23, hash: 20)	22
	SPA	63 (header: 23, hash: 40)	44
	MBSA	43 (header: 23, MAC: 20)	90
	BFBSA	23 (header: 23)	110
2.70 × 10^−4^ (Fair)	Secure Deluge	43 (header: 23, hash: 20)	22
	SPA	63 (header: 23, hash: 40)	44
	MBSA	43 (header: 23, MAC: 20)	90
	BFBSA	23 (header: 23)	92
6.51 × 10^−4^ (Bad)	Secure Deluge	43 (header: 23, hash: 20)	22
	SPA	63 (header: 23, hash: 40)	22
	MBSA	43 (header: 23, MAC: 20)	72
	BFBSA	23 (header: 23)	56

**Table 3:** Comparisons of Source Authentication Schemes.

Metric	Secure Deluge	SPA	MBSA	BFBSA
Authenticity	yes	yes	yes	yes
DoS attacks	weak	weak	robust	robust
Node capture attacks	robust	robust	weak	robust
Packet size	fixed	variable	fully variable	fully variable
Computation overhead	low	low	low	high
Communication overhead	high	modest	lowest	low
Storage overhead	low	low	low	high

## References

[1] Dutta P.K., Hui J.W., Chu D.C., Culler D.E. Securing the deluge network programming system. Proceedings of the 5th International Conference on Information Processing in Sensor Networks.

[2] Hyun S., Ning P., Liu A., Du W. Seluge: Secure and dos-resistant code dissemination in wireless sensor networks. Proceedings of the 7th International Conference on Information Processing in Sensor Networks.

[3] Lanigan P.E., Gandhi R., Narasimhan P. Sluice: Secure dissemination of code updates in sensor networks. Proceedings of the 26th IEEE International Conference on Distributed Computing Systems (ICDCS 2006).

[4] Tan H., Ostry D., Zic J., Jha S. (2013). A confidential and dos-resistant multi-hop code dissemination protocol for wireless sensor networks. Comput. Secur..

[5] Zhang R., Zhang Y. Lr-seluge: Loss-resilient and secure code dissemination in wireless sensor networks. Proceedings of the 31st International Conference on Distributed Computing Systems (ICDCS).

[6] He D., Chen C., Chan S., Bu J. (2012). Dicode: Dos-resistant and distributed code dissemination in wireless sensor networks. IEEE Trans. Wirel. Commun..

[7] Tan H., Zic J., Jha S.K., Ostry D. (2011). Secure multihop network programming with multiple one-way key chains. IEEE Trans. Mob. Comput..

[8] He D., Chen C., Chan S., Bu J. (2012). SDRP: A secure and distributed reprogramming protocol for wireless sensor networks. IEEE Trans. Ind. Electron..

[9] Deng J., Han R., Mishra S. Efficiently authenticating code images in dynamically reprogrammed wireless sensor networks. Proceedings of the Fourth Annual IEEE International Conference on Pervasive Computing and Communications Workshops (PERCOMW’06).

[10] Bohli J.-M., Hessler A., Maier K., Ugus O., Westhoff D. (2011). Dependable over-the-air programming. Ad Hoc Sens. Wirel. Netw..

[11] Lee J., Kim L., Kwon T. (2016). Flexicast: Energy-efficient software integrity checks to build secure industrial wireless active sensor networks. IEEE Trans. Ind. Inf..

[12] Chen D., He D., Chan S. (2015). Security-enhanced reprogramming with xors coding in wireless sensor networks. Information and Communications Security.

[13] Xie M., Bhanja U., Wei G., Ling Y., Hassan M.M., Alamri A. (2015). Secnrcc: A loss-tolerant secure network reprogramming with confidentiality consideration for wireless sensor networks. Concurr. Comput. Pract. Exp..

[14] Perrig A., Szewczyk R., Tygar J.D., Wen V., Culler D.E. (2002). Spins: Security protocols for sensor networks. Wirel. Netw..

[15] Dong W., Chen C., Liu X., He Y., Liu Y., Bu J., Xu X. (2014). Dynamic packet length control in wireless sensor networks. IEEE Trans. Wirel. Commun..

[16] Wei D., Yunhao L., Zhiwei Z., Xue L., Chun C., Jiajun B. (2014). Link quality aware code dissemination in wireless sensor networks. IEEE Trans. Parallel Distrib. Syst..

[17] Wei D., Jie Y., Pingxin Z. (2015). Exploiting error estimating codes for packet length adaptation in low-power wireless networks. IEEE Trans. Mob. Comput..

[18] Kim D., An S. Source authentication schemes for reprogramming with variable packet size in wireless sensor networks. Proceedings of the 12th Annual IEEE International Conference on Sensing, Communication, and Networking (SECON).

[19] Hui J.W., Culler D. The dynamic behavior of a data dissemination protocol for network programming at scale. Proceedings of the 2nd International Conference on Embedded Networked Sensor Systems.

[20] Wander A.S., Gura N., Eberle H., Gupta V., Shantz S.C. Energy analysis of public-key cryptography for wireless sensor networks. Proceedings of the Third IEEE International Conference on Pervasive Computing and Communications.

[21] Rozenblit M. Secure Software Distribution. Proceedings of the IEEE Network Operations and Management Symposium.

[22] Ramalingam K.C., Subramanian V., Uluagac A.S., Beyah R. Simage: Secure and link-quality cognizant image distribution for wireless sensor networks. Proceedings of the Global Communications Conference (GLOBECOM).

[23] Vuran M.C., Akyildiz I.F. Cross-layer packet size optimization for wireless terrestrial, underwater, and underground sensor networks. Proceedings of the 27th Conference on Computer Communications INFOCOM 2008.

[24] Bloom B.H. (1970). Space/time trade-offs in hash coding with allowable errors. Commun. ACM.

[25] Tarkoma S., Rothenberg C.E., Lagerspetz E. (2012). Theory and practice of bloom filters for distributed systems. IEEE Commun. Surv. Tutor..

[26] Ren K., Yu S., Lou W., Zhang Y. (2009). Multi-user broadcast authentication in wireless sensor networks. IEEE Trans. Veh. Technol..

[27] IEEE Standard for Information Technology—Local and Metropolitan Area Networks—Specific Requirements—Part 15.4: Wireless Medium Access Control (Mac) and Physical Layer (Phy) Specifications for Low Rate Wireless Personal Area Networks (Wpans). http://ieeexplore.ieee.org/xpl/login.jsp?tp=&arnumber=1700009&url=http%3A%2F%2Fieeexplore.ieee.org%2Fiel5%2F11161%2F35824%2F01700009.

[28] Polastre J., Szewczyk R., Culler D. Telos: Enabling ultra-low power wireless research. Proceedings of the Fourth International Symposium on Information Processing in Sensor Networks.

[29] Lee H., Choi Y., Kim H. (2007). Implementation of tinyhash based on hash algorithm for sensor network. World Acad. Sci. Eng. Technol. Int. J. Comput. Energ. Electron. Commun. Eng..

[30] Peter S., Langendorfer P., Piotrowski K. (2008). Public key cryptography empowered smart dust is affordable. Int. J. Sens. Netw..

